# The Detection of Alkaline Phosphatase Using an Electrochemical Biosensor in a Single-Step Approach

**DOI:** 10.3390/s91108709

**Published:** 2009-10-30

**Authors:** Joanne H. Wang, Kevin Wang, Brandon Bartling, Chung-Chiun Liu

**Affiliations:** 1 Department of Biology, Brown University, 171 Meeting Street, Providence, RI 02912, USA; E-Mail: jowang89@gmail.com (J.H.W.); 2 College of Medicine, University of Cincinnati, 231 Albert B. Sabin Way, Cincinnati, OH 45267, USA; E-Mail: wangkv@mail.uc.edu (K.W.); 3 Department of Chemical Engineering, Case Western Reserve University, 10900 Euclid Avenue, Cleveland, OH 44106, USA; E-Mail: bxb79@case.edu (B.B.)

**Keywords:** alkaline phosphatase, electrochemical detection, biosensor

## Abstract

A one-step, single use, disposable Alkaline Phosphatase (ALP) biosensor has been developed. It is based on the detection of phenol produced by an ALP enzymatic reaction. It can operate at 25 °C in a pH 10 medium. It measures ALP of 0–300 IU/L. The permissible concentrations of glucose, ascorbic acid and urea without interference are 10 mM/L, 5 mg/L and 400 mg/L, respectively. Experimental results are compared to those obtained by spectrophotometric measurements in bovine serum. Excellent linearity between the biosensor outputs and the ALP concentrations exists. The agreement between the measurements of this biosensor and the spectrophotometer is also outstanding.

## 1. Introduction

Alkaline phosphatase (ALP) is an enzyme that is present in human tissues in low but significant levels. ALP is present in high concentrations in parts of the body such as bone, liver, kidney, intestines, and the placenta [1]. The concentration of ALP can be used as a biomarker of diseases related to these areas. A normal level of ALP in the human blood generally ranges from 25 to 100 IU/L. However when the concentration of ALP is higher than 300 IU/L, it is an indicator of several diseases including liver diseases, liver cancer, hepatitis, bone disease, osteoblastic bone cancer, kidney cancer, and other ailments [2,3]. Heart failure, heart attack, and serious infection can also raise the ALP level [4]. On the other hand, low concentration of ALP is an indicator of malnutrition. In addition to diagnosing ailments, monitoring ALP level is useful in surveying the liver when medication dangerous to the liver are taken, and the same is also true in monitoring the effectiveness of treatment for the mentioned liver diseases.

Measurement of ALP level in physiological fluids is generally accomplished using spectrometric, spectrophotometric, or electrochemical detection techniques. The current clinical method of quantifying ALP is the spectrophotometric method based on a procedure published by McComb *et al.* [3] which catalyzes the transphosphorylation of *p*-nitrophenylphosphate (p-NPP) to *p*-nitrophenol (p-NP) in the transphosphorylating buffer. The reaction is enhanced through the use of magnesium and zinc ions. The change in absorbance at 405nm due to the formation of p-NP is directly proportional to the ALP activity.

Each of these detection methods has its merits and limitations. Spectrometric methods are a highly accurate method of determining the composition of a species based on mass and charge. They are able to resolve elemental composition of a variety of compounds including proteins and proteases to a very high degree [5,6]. Unfortunately, the high cost of equipment and low sample processing speed means this method is more suitable for research settings than point of care testing. The established method of detecting ALP uses spectrophotometric methods. Spectrophotometric methods have proven to be very dependable as evident by their wide adoption for research and clinical application [7], but they suffer from the requirements of expensive, physically large instruments and larger required sample volumes. While suitable for a clinical setting, the ability to use spectrophotometric methods at the point of care or in a field application is infeasible. Additionally, the advantage of electrochemical detection of ALP over traditional spectrophotometric detection methods has been studied by Thompson *et al.* [8]. The amperometric method has a detection limit of 7 nM for the product of the enzyme reaction, which is almost 20 times better than the spectrophotometric method. Similarly, with a 15-min reaction at ambient temperature and in a reaction volume of 1.1 mL, 0.05 μg/L ALP could be detected electrochemically, almost an order of magnitude better than by absorption spectrophotometry. An alternative ALP detecting method combines electrochemical detection methods with other instruments. Capillary electrophoresis with an electrochemical detection method for ALP measurement has been examined by Jiao *et al.* [9]. While electrophoresis provides good sensitivity to ALP detection, it still requires a large analytical instrument. An ideal detection system would involve a relatively small-scale, portable, and disposable sensor system. Research attempts have been made to develop the ideal sensor for ALP detection. The immobilization of horseradish peroxidase and an anti-thyrotropin monoclonal antibody to the surface of an activated carbon electrode by simple passive absorption in combination with amperometric detection has been used by Ho *et al.* to detect ALP levels [10]. Similarly, a modified graphite electrode with an enzyme tyrosinase immobilized in a Nafion membrane was developed by Nistor *et al.* [11]. The works by Nistor and Ho both showed that a small form factor biosensor was suitable for the detection of ALP. However, the methods used involved immobilization of enzymes and antibodies on the electrodes which could be technically difficult, and the shelf-life of the electrode with the enzymes immobilized onto its surface could also become an issue [12,13]. The inclusion of the filtering membrane into the design also introduced increased diffusional resistances and complexity to the overall sensor design.

**Scheme 1.**
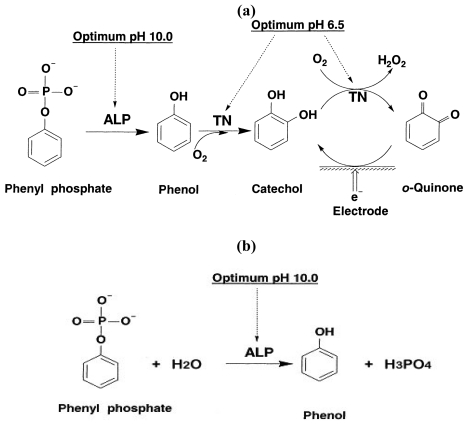
(a) Schematic of the two-step reaction to detect ALP (b) The simple one-step reaction required for the electrochemical detection of ALP.

A two-step, dual-enzyme detection method to indirectly measure ALP concentrations based on the electrochemical oxidation of generated *o*-quinone from the commercially available phenyl phosphate has been developed by Ito *et al.* [14]. This two-step reaction sequence is shown in Scheme 1a.

While this approach appears to be feasible, the detection process is complicated. The process requires an additional enzyme and operates at two different pH conditions. In the first step, the reaction is carried out in a medium of pH 10. In the second step, the reaction is carried out in a medium of pH 6.5. This change in the pH value of a test medium is impractical for the detection of ALP. However, this two-step reaction suggests that under the appropriate conditions, ALP can be quantified by measuring phenol produced in the first step of this enzymatic reaction route. Thus, the detection of ALP would be greatly simplified, if an electrochemical sensor could detect phenol directly. Bauer *et al.* [15] report a phenol-indicating biosensor to quantify ALP and describe the bienzyme biosensor operating in a flow injection system. This biosensor consists of a Clark-type oxygen sensor with a membrane on which both tyrosinase and quinoprotein glucose dehydrogenase are immobilized. The phenol produced is then oxidized within the membrane by tyrosinase to *o*-quinone via catechol synthesis. The oxygen consumption is then used to quantify the phenol produced, which indicated the concentration of the ALP present. The requirement of two enzymes and a membrane-covered oxygen sensor of this approach are relatively complicated and impractical.

Metallic nano-particles deposited onto the surface of activated carbon can serve as an excellent catalyst for reactions including enzymatically-produced electrochemically active species, such as H_2_O_2_ and others. The favorable surface area to mass ratio leads to the exploitation of the use of nano-particle noble metal catalysts for biosensor development. In our past studies, a 5% by weight of iridium nano catalyst is added into activated carbon powders formulating a screen printable ink for the manufacturing of the biosensor prototypes. The details of the formulation of the ink, the physical dimensions, and the manufacturing process of the biosensor prototype have been given elsewhere [16-19]. The incorporation of the nano-metallic catalyst can lead to the use of a lower oxidation or reduction potential of the produced electrochemically active species. Consequently, this may minimize potential interference by the oxidation or reduction of other species at a higher electrochemical potential.

Enzymatic reactions which produce H_2_O_2_ and NADH (nicotinamide adenine dinucleotide, reduced form) have been used effectively for the detection of various biomarkers using the iridium nano catalyst contained platform biosensor [16-19]. Phenol is considered to be an electrochemically active species. Thus, if the iridium nano particle contained biosensor prototype can be used to detect phenol specifically in a physiological fluid, a single-use, disposable ALP biosensor based on a single reaction step as shown in Sheme 1b can then be realized.

**Figure 1.**
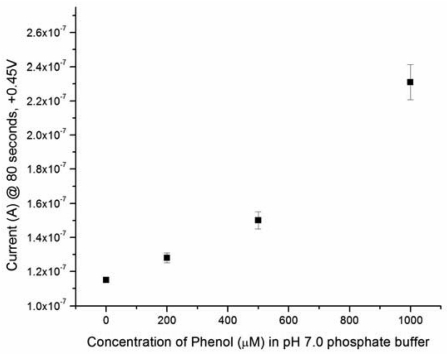
Amperometric phenol detection in a standard pH 7.0 phosphate buffer solution with 150 mM KCl supporting electrolyte. Points represent average values from multiple experiments for n ≥ 3 samples each. Error bars represent the relative standard deviation of the measurement.

Thus, the objective of this research endeavor is to develop a single use, disposable ALP biosensor which can be produced inexpensively and is simple to operate. As mentioned, this biosensor is based on the detection of phenol effectively in the first step of the enzymatic reaction route as shown in Figure 1b, and this single use, disposable ALP biosensor can be produced inexpensively using thick film screen printing processing. In this study, the experimental assessment of the performance of this ALP biosensor has been carried out. The temperature effect and potential interference studies have also been undertaken and will be discussed. The experimental results of this biosensor in calf bovine serum were compared with those from the “gold standard” spectrophotometric measurement obtained in a clinical laboratory, and the results are in excellent agreement. Hence, this single use, disposable ALP biosensor represents a unique and simple detection method for ALP in bovine serum leading to potentially extensive application for ALP quantification.

## 2. Results and Discussion

As described, this iridium nano-particle modified single use, disposable biosensor prototype has shown excellent performance for the detection of electrochemically active species, such as H_2_O_2_, NADH and others. Phenol is considered an electrochemically active species. However, the use of direct phenol detection as a means to quantify the analyte or the enzyme in an enzymatic reaction route has not been fully explored. In the reactions shown in Scheme 1a,b, it is obvious that if a biosensor can detect phenol specifically, as shown in Scheme 1b, the detection of ALP can be greatly simplified. Thus, the first essential study would be to assess the feasibility of detecting phenol in a typical test medium, such as phosphate buffer solution (PBS) using the iridium-modified biosensor prototype. In this study, PBS at pH = 7.0 was used as the test medium. Phenol in the concentration range of 0 to 1,000 μM was used in this study. Solutions of phenol in buffer were prepared prior to testing. A 5 μL droplet of the solution was placed onto a sensor, held at room temperature (∼25 °C), that was connected to a potentiostat. The working electrode was held at +0.45 V, versus Ag/AgCl reference electrode, while the current was recorded over time.

Figure 1 shows the linear relationship between the iridium nano-particle modified single use, disposable biosensor and the phenol concentration over the range of 0 to 1,000 μM in a pH 7.0 PBS buffer at ambient temperature. Experimental measurements were made in triplicates or more at each phenol concentration. The experiment results ensured the feasibility of measuring phenol using this biosensor prototype. Consequently, the measurement of ALP levels can be assessed by detecting levels of generated phenol. This would be consistent with the reaction scheme shown in Scheme 1b.

It should be recognized that there were other chemical species used in this study. It would be essential to assess that the chemical species would not contribute to the sensor output of this biosensor prototype. Thus, a methodical examination of the other added chemicals used in the experiment were studied to determine potential electrochemical interference.

**Figure 2.**
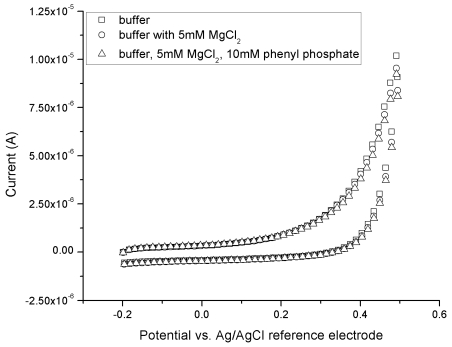
Cyclic-voltammetric scans from –0.2 V to +0.5 V at a scan rate of 10 mV/s in a pH 10 PBS with 150 mM KCl supporting electrolyte.

Cyclic-voltammetric scans were conducted using a pH 10 PBS buffer with 150 mM of KCl supporting electrolyte. The increase in pH of the buffer was necessary in order to enhance the enzymatic phosphorylation of the phenyl phosphate by ALP. The scan was from –0.2 V to +0.5 V at a scan rate of 10 mV per second for a total of eight cycles. Subsequent scans were conducted, using fresh sensors, but with the addition of MgCl_2_ and phenyl phosphate to the buffer. It can be seen in Figure 2 that over the range studied no increased current is generated beyond that of the background. This gives confirmation that the addition of MgCl_2_ and phenyl phosphate to the solution did not increase the background current at the levels used.

### 2.1. Temperature effect on the ALP biosensor performance

It was recognized that this ALP biosensor would be affected by the operating temperature of the test environment. It was also anticipated that at elevated temperatures close to the human body temperature, i.e. approximately 37 °C, the ALP biosensor would perform better compared to lower temperature. In this study, the performance of this ALP biosensor was experimentally evaluated at three different temperatures, i.e. ambient temperature (23–25 °C), 32 °C, and 37 °C. As expected, the current output of the ALP biosensor increased at higher operating temperatures. However, in terms of practical application, if the biosensor could operate at ambient temperature it would simplify the electronic interface and the instrumentation of this ALP biosensor system. We found that this ALP biosensor could operate at ambient temperature if the quantity of phenyl phosphate, the co-reactant in this reaction, was sufficient so as not to become a rate-limiting controlled co-reactant at ambient temperature. The experimental results will be discussed further later.

### 2.2. Evaluation of potential interference of other components in the test medium

**Figure 3.**
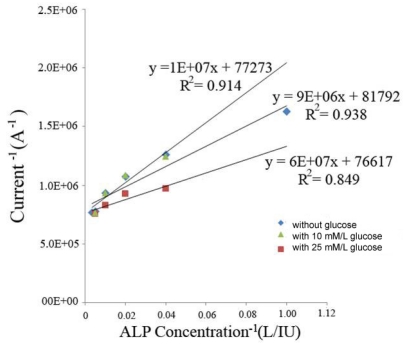
Sensor response to the detection of ALP with, and without, glucose present.

There are commonly existing biological components which may affect the performance of the ALP biosensor. For example, the presence of glucose, ascorbic acid and urea may affect the performance of this biosensor. In human blood, glucose levels of 25 mM/L or greater will result in a severely ill individual. A concentration of 10 mM/L in human blood is still considered dangerously high [17]. Thus, if the sensor is capable of performing in concentrations at these levels, it is safe to assume they will perform in standard samples. This ALP biosensor appeared to perform well within the glucose concentration of 10 mM/L or lower. Figure 3 shows the biosensor output in the presence of 10 mM/L and 25 mM/L of glucose in the presence of ALP. It could be seen in Figure 3 that the presence of the glucose at a 10 mM/L concentration level did not significantly affect the biosensor output. On the other hand, the presence of 25 mM/L of glucose in solution showed interference of the ALP biosensor. We recognize that glucose concentrations higher than 10 mM/L will cause interference on the ALP sensor measurement. However, as mentioned, at a glucose level of 10 mM/L or higher the patient would be already seriously ill.

**Figure 4.**
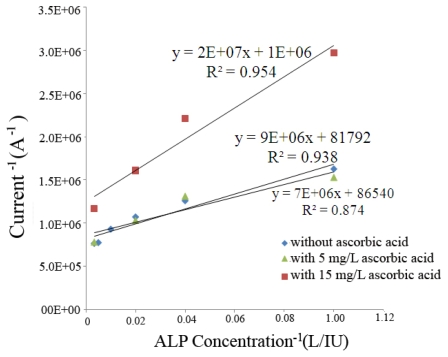
Sensor response to the detection of ALP with, and without, ascorbic acid present.

The potential interference of ascorbic acid to the ALP biosensor was also investigated in this study. As another potential interfering species in the body, concentrations of 5 and 15 mg/L of ascorbic acid were studied. A concentration of 5 mg/L of ascorbic acid in human blood represents a value in the upper range of that expected for a normal individual [20]. While a concentration of 15 mg/L of ascorbic acid reflects a concentration that would be considered beyond that experienced in a normal individual [20]. Similar to the observations made in the glucose interference study, at an ascorbic acid concentration of 15 mg/L or higher, interference of the ALP biosensor does occur. However, at the lower ascorbic acid concentration of 5 mg/L the effect of the ascorbic acid to the ALP biosensor performance is rather minimal, as shown in Figure 4.

**Figure 5.**
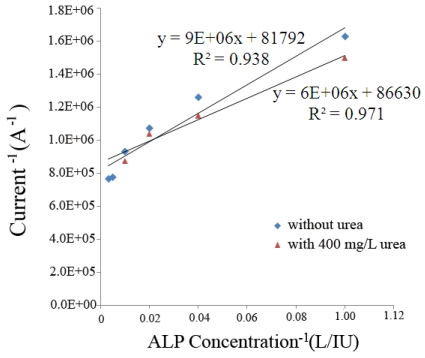
Sensor response to the detection of ALP with, and without, uric acid present.

The last chemical that was tested for its interference contribution was urea. High concentrations of urea are found in the body at 400 mg/L [20]. Figure 5 shows the relationship between current and concentration of ALP with and without 400 mg/L of urea.

It appeared that high concentrations of urea did not significantly affect the result. Thus interference of the biosensor in the presence of glucose, ascorbic acid and urea concentrations, higher than 10 mM/L, 5 mg/L, and 400 mg/L respectively, will occur. This is a limitation of this ALP biosensor.

### 2.3. Calibration of the ALP Biosensor in Bovine Serum

**Figure 6.**
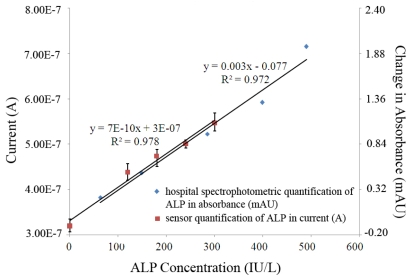
Comparison of measurements made using a Dimension® RxL Max™ spectrophotometer, change in absorbance (mAU), to the amperometric sensor response current (A) taken at 600 seconds.

The calibration and the assessment of the ALP biosensor were carried out in bovine serum over the ALP concentration range of 0–300 IU/L. The amperometric current was compared with the results obtained by spectrophotometer measurements of a Dimension® RxL Max™ spectrophotometer which is a gold standard technique for ALP measurement. Figure 6 shows the result of the biosensor and the spectrophotometer measurements. An excellent linear relationship between the biosensor output and ALP concentration in bovine serum exists. The output of the ALP biosensor and the spectrophotometric measurements are in excellent agreement, confirming that the ALP biosensor can be used effectively in bovine serum.

## 3. Experimental Section

### 3.1. Chemicals and Solutions

A solution of MgCl_2_ (Sigma-Aldrich, St. Louis, MO) and the pH 10 phosphate buffer solution (PBS) (Sigma-Aldrich) was prepared with a 5 mM concentration of magnesium chloride. MgCl_2_ provided Mg^2+^, which functioned as a cofactor for the ALP (Sigma-Aldrich) in the solutions [18]. A separate stock solution was also prepared using PBS, 5mM MgCl_2_ and 10 mM phenyl phosphate (Sigma-Aldrich). For the interference assessment, glucose (Sigma-Aldrich), ascorbic acid (Fisher Scientific, Hampton, NH), and urea (Sigma-Aldrich) were used. Bovine serum samples were purchased from Invitrogen (San Diego, CA). ALP was obtained from USB (Cleveland, OH).

### 3.2. Instrumentation and Electrochemical Sensor

An Electrochemical Workstation CH Instrument model 660C (Austin, TX) was used to carry out cyclic voltammetric and amperometric studies. The sensor output was recorded in a PC that was connected to the Workstation using a standard edge clip connection.

Measurements were made at 23–25 °C, 32 and 37 °C. The sensor was placed on top of aluminum foil shaped into a small dome that was secured over a temperature controlled water bath, this was done to maintain a constant temperature. Measurements of the temperature effect on the ALP biosensor were then carried out.

As described, this *in vitro*, single-use, disposable biosensor prototype was manufactured by thick-film screen printing processing. The sensor contained an Ir-modified carbon working and an Ir- modified counter electrode and an Ag/AgCl reference electrode. The biosensor prototypes were printed on a polyester substrate, and the working electrode had a total surface area of 7.85 × 10^−3^ cm^2^. Details of the fabrication processing and the physical configuration of this biosensor prototype have been given elsewhere [16-19]. However, it is important to recognize that the iridium nano-particle-containing ink needed to be screen printable. Thus, the composition of the iridium nano-particles, the binder and the solvent chosen are important. In our study, an ink-based solution was first prepared. Typically, 10 mL pH 7.0 phosphate buffer solution was mixed with 1.36 mL polyethylenimine and 0.34 g of 2-hydroxy- cellulose resulting in a screen-printable ink solution. Ir-carbon particles (5% Ir by weight) obtained from BASF (Florham Park, NJ, USA) was used. Details of the preparation and the fabrication steps of the Ir-contained carbon working electrode for this single use, disposable biosensor have been given elsewhere [16-19]. The nano-particle metallic catalyst was used to enhance the sensitivity of the biosensor to the detection of the enzymatically generated phenol.

### 3.3. Testing Procedure

The validity and applicability of this iridium nano-catalyst contained biosensor for ALP detection had to be experimentally verified and assessed. Therefore, the experimental protocols established in this study included the following investigations:

1. Evaluation of the potential effects of the chemical species involved in the enzymatic reaction.
  As shown in Scheme 1b and other information, the preliminary studied enzymatic reaction was carried out first in a phosphate buffer solution (PBS) at pH = 7.0 in order to determine its feasibility for ALP detection by quantifying the produced phenol. This reaction involved MgCl_2_ and phenyl phosphate which were needed to activate the ALP enzymatic reaction and served as the reaction substrate, respectively. It was necessary to assess if any of these basic materials, the PBS, the MgCl_2_ and the phenyl phosphate may contribute to the sensor output in ALP detection. Cyclic voltammetric measurements were carried out in this experimental evaluation.
2. Sensitivity of phenol detection using the biosensor prototype.
  It would be essential that this iridium nano-catalyst contained biosensor prototype could be used effectively for the detection of phenol as a means of quantifying ALP in a surrogate fluid. This assessment was first undertaken in PBS at a fixed pH value. Both cyclic voltammetric and amperometric studies were carried out. The cyclic voltammetric study assessed the appropriate oxidation potential of phenol, whereas the amperometric measurements aided in the assessment of the relationship between the sensor output (in current) and the ALP concentration level in a test medium. The ALP concentration range of 0 to 300 IU/L used covered the important physiological range of ALP in a biological system.
3. Effect of operating temperature on the performance of the ALP biosensor.
  The operating temperature can affect the performance and the sensor output of the ALP biosensor directly. It was anticipated that at a higher operating temperature the sensor current would increase. However, a biosensor operating at a temperature higher than ambient temperature will require an additional heating element. This would complicate the single use, disposable ALP biosensor design. In this study, the temperature effect on the performance of the ALP biosensor was experimentally assessed at three temperatures, ambient temperature (approximately 23–25 °C), 32 and 37 °C. As mentioned, a constant temperature water bath was used in this study to maintain temperature.
4. Evaluation of potential interference by the biological species.
  Selected biological species, such as glucose, ascorbic acid and uric acid can affect ALP detection. In this study, a stock solution containing 25 mM/L of glucose was prepared and added to a test solution containing ALP in the range of 10–300 IU/L. Similarly, a stock solution of 15 mg/L of ascorbic acid solution, and uric acid stock solution of 400 mg/L were prepared for the potential interference studies. The concentration levels of these stock solutions chosen were based on the maximum reported values for interfering species found in physiological fluid.
5. Evaluation of the performance of this ALP biosensor in bovine serum.
  In order to assess the sensor's ability to detect ALP in bovine serum, test samples containing varying concentrations of ALP were tested with the biosensor and compared to measurements obtained by a Dimension® RxL Max™ spectrophotometer (Siemens Healthcare Diagnostics, Inc., Tarrytown, NY). This was done to establish a comparison between the traditional method of detection and the sensor's performance. Testing samples containing shrimp alkaline phosphatase (USB, Cleveland, OH) were diluted in bovine serum over the concentration range of 0 to 300 IU/L. The ALP levels of the samples were then measured by both the spectrophotometer and our electrochemical based biosensor. The samples were stored at –20 °C when they were not used. Electrochemical testing of the ALP biosensors was conducted following the following steps:
  1. 75 μL of the ALP contained bovine serum was added into a 0.6 mL microtube along with 75 μL of pH 10 phosphate buffer solution containing 150 mM of phenyl phosphate.
  2. The tube was vortexed for 5–10 seconds to ensure a complete mixing.
  3. 5 μL of the combined solution was pipetted onto the surface of the ALP biosensor.
  4. The potential was set at +0.45 V vs Ag/AgCl reference electrode, for a time of 600 seconds and was operated at 25 °C.

The ALP containing samples for the spectrophotometric measurement were prepared identical to those for the biosensor study, and the operation of the spectrophotometer followed the standard instruction of the instrument.

## 4. Conclusions

Electrochemical detection of ALP traditionally requires a two-step reaction, involving a change in pH from 10 to 6.5 of the testing sample. In this study, a simplified process of ALP detection was proposed using a single step approach. ALP's substrate, phenyl phosphate was enzymatically decomposed to phenol, which was oxidized electrochemically. A three-electrode sensor prototype was used to quantify the oxidation current produced. Quantification of the current could be related to original solution ALP concentrations.

The feasibility and validity of detecting phenol using this ALP biosensor was carried out, and the results were very good at an oxidation potential of +0.45 V versus Ag/AgCl reference electrode. Cyclic voltammetric studies in solution with 150 mM KCl as supporting electrolyte, buffer solution with 5 mM MgCl_2_ and the buffer solution with 5 mM MgCl_2_ with 10 mM phenyl phosphate were carried out. This gave confirmation that addition of MgCl_2_ and phenyl phosphate to solution did not increase the background current ensuring any current measured would be derived only from the electrochemical oxidation of phenol produced by the ALP enzymatic reaction. Operational temperature effects at 25, 32 and 37 °C were evaluated. At a higher temperature, the biosensor output increased as anticipated. The biosensor was shown to be able to operate at ambient temperature, and for simplicity and practical use the biosensor was operated at ambient temperature in subsequent studies of the assessment of the sensor performance. Potential chemical interference of the sensor performance was investigated. It was found that only minute interference would occurred, if the of glucose, ascorbic acid and urea concentrations were higher than 10 mM/L, 5 mg/L, and 400 mg/L respectively. Physiological important ALP concentrations levels (0–300 IU/L) in bovine serum were successfully detected by this single-step method using a single use, disposable biosensor. The performance of the ALP biosensor was assessed by comparing it to the measurements made by a gold standard method in ALP detection, spectrophotometry. A linear relationship was established between current at 600 seconds and ALP concentration with the ALP biosensor, and an excellent agreement was established between the change in absorbance of the spectrophotometric measurements and the current output of the ALP biosensor in bovine serum over the ALP concentration range of 0–300 IU/L. This comparison supported that this single-step, single use, disposable ALP biosensor could be used effectively to evaluate ALP levels in bovine serum.
